# Eight-Week Mindfulness Training Effectively Improves Performance in Children with ADHD: A Comparison with Cognitive-Behavioral Training

**DOI:** 10.3390/bs16071128

**Published:** 2026-07-06

**Authors:** Chenguang Zhao, Yifei Sun, Wei Zhang

**Affiliations:** School of Psychology, Central China Normal University, Wuhan 430079, China

**Keywords:** ADHD, mindfulness training, cognitive-behavioral training, test anxiety, self-concept

## Abstract

Background: This study compared the effects of mindfulness training (MT) and cognitive-behavioral training (CBT) on ADHD symptoms, test anxiety, and self-concept in children with ADHD and explored potential pathways of change associated with the two interventions. Methods: After a two-stage screening and eligibility confirmation procedure, 63 children with ADHD were included in the final analyses, including 19 in the MT group, 25 in the CBT group, and 19 in the control group. The MT and CBT groups received an eight-week intervention, whereas the control group continued regular classroom activities. ADHD symptoms, test anxiety, and self-concept were assessed before and after the intervention. Data were analyzed using 3 × 2 repeated-measures ANOVA and exploratory cross-lagged analyses. Results: Significant Group × Time interactions were found for ADHD symptoms, F(2, 60) = 29.74, *p* < 0.001, *η_p_*^2^ = 0.5; test anxiety, F(2, 60) = 7.77, *p* = 0.001, *η_p_*^2^ = 0.21; and self-concept, F(2, 60) = 18.9, *p* < 0.001, *η_p_*^2^ = 0.39. Simple effects analyses showed that both the MT and CBT groups showed significant reductions in ADHD symptoms and test anxiety and significant improvements in self-concept, whereas the control group showed no significant pretest-to-posttest changes. Exploratory cross-lagged analyses showed different patterns of association between ADHD symptoms and test anxiety in the two intervention groups. Conclusions: Both MT and CBT were associated with improvements in ADHD symptoms, test anxiety, and self-concept in children with ADHD. The exploratory pathway findings suggest that the two interventions may be linked to partly different patterns of change. However, further studies are needed to verify these preliminary findings.

## 1. Introduction

Attention-deficit/hyperactivity disorder (ADHD) is a neurodevelopmental disorder characterized by features of inattention, hyperactivity, and impulsivity (American Psychiatric Association, 2013). ADHD is commonly identified during childhood, with epidemiological studies suggesting that diagnosed ADHD is particularly prevalent among school-aged children, including those around 9 years of age (Danielson et al., 2018; Erskine et al., 2013; Song et al., 2021). Children with ADHD often experience difficulties in emotional regulation, academic performance, peer relationships, and behavioral adjustment (Faraone et al., 2024; Sayal et al., 2018). These challenges may place substantial demands on families, schools, and healthcare systems, contributing to increased educational, healthcare, and family-related costs (Chorozoglou et al., 2015; Doshi et al., 2012; Snell et al., 2013). These findings highlight the need for effective interventions to improve behavioral and psychosocial outcomes in children with ADHD.

### 1.1. Cognitive-Behavioral Training and Mindfulness Training for Children with ADHD

The intervention and treatment of ADHD have consistently been at the forefront of attention in the field. Non-pharmacological treatments, such as Cognitive-Behavioral Training (CBT) and Mindfulness Training (MT), are currently highly favored due to their low cost, minimal side effects, and absence of drug dependence risks, especially in China (Li et al., 2019; Moore et al., 2019; Nimmo-Smith et al., 2020). CBT aims to enhance mental health by adjusting maladaptive cognitions and providing behavioral training (Groenman et al., 2022; Lambez et al., 2020; Y. P. Zhang & Liu, 2005). MT refers to a way of experiencing the present moment without judgment, emphasizing focused attention on the present and non-judgmental acceptance (Cardaciotto et al., 2008; Kabat-Zinn, 2003; Zheng et al., 2022; Zhou et al., 2021).

Existing evidence suggests that CBT and MT may improve ADHD-related difficulties through partly different intervention targets. CBT has been widely applied in ADHD interventions and appears particularly relevant to reducing core ADHD symptoms, externalizing behaviors, and behavioral regulation difficulties (Riise et al., 2021; Young et al., 2020). For example, previous studies have reported that CBT can reduce aggressive behavior and improve attention deficits and hyperactivity in children with ADHD (Gould et al., 2018; Vacher et al., 2022). MT, by contrast, has been more closely associated with improvements in attentional awareness, self-control, emotional regulation, and cognitive functioning (Lee et al., 2022; Robe & Dobrean, 2023; Van de Weijer-Bergsma et al., 2012). However, compared with the adult literature, empirical evidence on MT for children with ADHD remains relatively limited. Existing studies have reported perceived improvements in emotion regulation and cognitive functioning after mindfulness-based programs, but some were qualitative in nature or showed less consistent between-group effects at posttest (Siebelink et al., 2021, 2022). Taken together, these findings indicate that both CBT and MT may be beneficial for children with ADHD, but further research is needed to clarify their relative intervention effects and potential pathways of change.

### 1.2. Limitations of Previous Research

Based on the aforementioned studies, both CBT and MT appear to have beneficial effects for children with ADHD. However, important limitations remain in the existing literature. Some studies have used simple pre-post designs to examine the effects of mindfulness interventions but did not include control groups for comparison (Van de Weijer-Bergsma et al., 2012; D. Zhang et al., 2017). Other studies included control groups but often used no-treatment controls rather than placebo or active control conditions, which limits the strength of causal interpretation (Siebelink et al., 2021; Valero et al., 2022; Van der Oord et al., 2012). Therefore, further randomized controlled studies are needed to provide more rigorous evidence for the effects of MT in children with ADHD.

In addition, relatively few studies have directly compared MT and CBT within the same intervention framework for children with ADHD. Such a comparison is important not only because both interventions may be effective, but also because they may target partly different domains of functioning. Meta-analytic evidence suggests that CBT is effective in improving externalizing behaviors in children with ADHD, although its effects on other ADHD-related symptoms may be more limited (Lambez et al., 2020; Riise et al., 2021). By contrast, MT has been associated with reductions in anxiety and improvements in emotion regulation in individuals with ADHD (Cairncross & Miller, 2020; Miller & Brooker, 2017). According to the Monitor and Acceptance Theory, MT includes two core components: attention monitoring and acceptance. Attention monitoring enhances awareness of present-moment experience, whereas acceptance promotes a non-judgmental attitude toward internal experiences and may help reduce negative emotional responses (Lindsay & Creswell, 2017, 2019). From this perspective, MT and CBT may show different patterns of association with ADHD symptoms and related psychosocial outcomes. Therefore, comparing these two interventions may provide useful evidence for developing more targeted interventions for children with ADHD.

### 1.3. Test Anxiety and Self-Concept in Children with ADHD

In addition to core ADHD symptoms, test anxiety and self-concept are important psychosocial outcomes for children with ADHD (Dan & Raz, 2015; Nelson, 2013; Nelson et al., 2014). ADHD is often identified during the primary-school years, a developmental period in which children begin to face increasing academic demands and more frequent evaluations. In China, this stage often corresponds to the third or fourth grade of primary school, when learning tasks become more challenging and examinations become more frequent (Huang & Zhou, 2019). In this educational context, test anxiety may become a particularly relevant emotional outcome for children with ADHD. Self-concept is also important because it reflects how children evaluate their own abilities and personal worth, and it has been linked to academic performance, social relationships, and emotional well-being (Silvestri et al., 2018). Previous studies have shown that children with ADHD often report lower levels of self-concept than their typically developing peers (Houck et al., 2011; Nelson, 2013). Therefore, assessing both test anxiety and self-concept may provide a broader understanding of whether MT and CBT improve not only ADHD symptoms but also related emotional and self-evaluative functioning in children with ADHD.

In summary, the present study examined the effects of MT and CBT on ADHD symptoms, test anxiety, and self-concept in children with ADHD. Specifically, this study addressed two main questions. First, do MT and CBT improve ADHD symptoms, test anxiety, and self-concept compared with a control condition? Based on previous findings, we hypothesized that both MT and CBT would reduce ADHD symptoms and test anxiety and enhance self-concept. Second, do MT and CBT show different patterns of association among ADHD symptoms, test anxiety, and self-concept? Drawing on cognitive-behavioral theory and the Monitor and Acceptance Theory, we expected that CBT-related changes would be more closely associated with behavioral regulation of ADHD symptoms, whereas MT-related changes would be more closely associated with emotional regulation, particularly test anxiety.

## 2. Methods

### 2.1. Participants

Participants were recruited from primary schools in Hubei Province, China. A two-stage screening and confirmation procedure was used to identify eligible children. In the first stage, parents and teachers completed the DSM-5 ADHD symptom checklist and the Chinese version of the Swanson, Nolan, and Pelham Rating Scale, Version IV (SNAP-IV). Children who met the DSM-5 symptom threshold for ADHD and exceeded the established SNAP-IV cut-off score were considered potential participants (Zhou et al., 2013). In the second stage, these children were further evaluated by trained professionals through clinical interviews with the children and their parents. The final eligibility decision was based on the integration of DSM-5 symptom criteria, SNAP-IV scores, and clinical interview information. Thus, parent- and teacher-report measures were used for initial screening rather than as the sole basis for diagnosis.

During the clinical interview, children were excluded if they had a documented intellectual disability, autism spectrum disorder, current use of psychiatric medication that could substantially affect attention or behavioral performance, substance-related problems, or other severe psychiatric or medical conditions that could interfere with participation in the intervention.

Following the two-stage screening and eligibility confirmation procedure, 96 students were identified as eligible from the initial pool of 720 students. These eligible participants were randomly assigned to one of three groups, namely mindfulness training (MT), cognitive-behavioral training (CBT), or a control group. During the eight-week intervention period, 17 participants in the MT group and 16 participants in the CBT group withdrew or failed to complete the full study procedure, whereas no participants dropped out of the control group. Consequently, 63 students were included in the final statistical analyses. The final sample consisted of children aged 8 to 10 years (M = 8.4), including 33 boys and 30 girls. Specifically, there were 19 children in the MT group (mean age 8.53 ± 0.61), 25 in the CBT group (mean age 8.4 ± 0.58) and 19 in the control group (mean age 8.26 ± 0.56).

### 2.2. Procedure

The experiment employed a 3 (group: MT group, CBT group, control group) × 2 (test time: pretest, posttest) mixed experimental design. Eligible participants were randomly assigned to the MT, CBT, or control group using a computer-generated randomization sequence. The MT and CBT groups received one 45-min session per week for eight consecutive weeks, whereas the control group continued their regular classroom activities. To reduce contamination between groups, the MT and CBT sessions were conducted in separate classrooms, and participants from different intervention groups did not receive training together.

Given the nature of behavioral interventions, full double blinding of participants and intervention facilitators was not feasible. However, several procedures were used to reduce potential bias. Participants were not informed of the specific study hypotheses regarding the expected differences between MT and CBT. Outcome assessment and data entry were conducted using standardized procedures, and the researchers responsible for statistical analysis were not involved in delivering the interventions.

Intervention fidelity was addressed through several procedures. Both intervention programs followed structured session plans, and each session included standardized components, including warm-up, instruction, practice, and summary. The facilitators had prior professional training and experience in the relevant intervention approach. To ensure consistency across sessions, facilitators followed the intervention manuals and completed each session according to the planned content. Regular practice assignments were provided after each session, and facilitators reviewed participants’ practice and provided feedback in subsequent sessions. At the same time, the interventions were implemented in a natural school setting rather than in a laboratory context. Participation was voluntary rather than forced, and children could discontinue participation if they were unable or unwilling to continue.

### 2.3. Measures

Chinese version of Swanson, Nolan and Pelham, Version IV Scale-parent form (SNAP-IV). The SNAP-IV, designed by Swanson (1981) based on DSM diagnostic criteria, serves as a crucial tool for the screening and adjunct diagnosis of ADHD. The Chinese version of the SNAP-IV was translated by Zhou et al. (2013) and is available in both parent and teacher forms. The scale includes a total of 26 items, organized into three subscales: Attention Deficit Scale, Hyperactivity/Impulsivity Scale, and Oppositional Defiant Scale. Each item is rated on a four-point scale ranging from 0 to 3: 0 = no presence, 1 = a little, 2 = a moderate amount, and 3 = a great deal. The Cronbach’s α of the total scale is 0.95, and the subscale reliabilities are 0.90, 0.89, and 0.88, indicating good reliability and validity for the scale.

Test Anxiety Inventory (IAT): Test anxiety was measured using the Test Anxiety Inventory (IAT), designed by Spielberger (2010). The scale comprises a total of 20 items, encompassing two dimensions: Worry and Emotionality. Each item is rated on a four-point scale (1–4): 1 = Never, 2 = Sometimes, 3 = Often, and 4 = Always. A higher total score indicates stronger test anxiety. The Cronbach’s α = 0.9 and McDonald’s omega = 0.83, indicating good reliability and validity for the scale.

Children’s Self-Concept Scale (PHCSS): The Children’s Self-Concept Scale, developed by Piers-Harris (Piers, 1984), was designed to measure children’s self-concept. The scale consists of 80 items, including six subscales: Behavior, Intellectual and School Status, Physical Appearance and Attributes, Anxiety, Sociability, and Happiness and Satisfaction. Each item is scored by assigning 1 point for a “yes” response and 0 points for a “no” response. A higher total score indicates a stronger level of self-concept. The Cronbach’s α = 0.86 and McDonald’s omega = 0.81, indicating that the scale demonstrates good reliability and validity.

Mindfulness Training (MT): In this study, we designed a mindfulness training course, taking into account the intellectual and cognitive development characteristics of children. The mindfulness training program in this study was referenced from Li et al. (2019) and Xu et al. (2015). The course placed emphasis on stimulating students’ learning interests. Details of the course content are provided in Supplementary Table S1. The training sessions were conducted once a week, with each session lasting 45 min, and the entire program spanned eight weeks. In the course design, firstly, we used simple and vivid instructions to ensure that students could easily understand. Secondly, each session was given an interesting name (e.g., “Frog Meditation”) to attract the students’ interest. Lastly, a reward mechanism was incorporated into the training sessions to inspire students’ enthusiasm for active participation. Each session included four segments: warm-up, explanation, practice, and summary. The training content included mindfulness breathing, mindfulness observation, coping with negative emotions, and self-acceptance. Each exercise in the program was accompanied by a corresponding instructional audio guide. To ensure the effectiveness of the intervention, we assigned homework to students after each session. The facilitator emphasized the importance of regular practice in each session and provided timely feedback to address any issues encountered by students during their practice.

Cognitive-Behavioral Training (CBT): The CBT program in this study was referenced from Shi et al. (2019) and Y. P. Zhang and Liu (2005). Details of the CBT course content are provided in Supplementary Table S2. The training sessions were conducted once a week, with each session lasting 45 min, and the entire program spanned eight weeks. Similar to mindfulness training, each session included four segments: warm-up, explanation, practice, and summary. In previous research, cognitive-behavioral training for ADHD primarily included two major modules: cognitive adjustment and behavioral modification. Scholars mainly utilized self-guidance and impulse control for training (Lambez et al., 2020; Riise et al., 2021). Our study made slight modifications to the training program, taking into account the characteristics of children. In the self-guided training, children were guided to control their cognition and behavior using verbal instructions. In impulse control training, children were guided to practice pausing before speaking, waiting for their turn, and using verbal self-instruction to regulate impulsive responses. To ensure the effectiveness of the intervention, we assigned homework to students after each session. The facilitator emphasized the importance of regular practice in each session and provided timely feedback to address any issues encountered by students during their practice. The MT and CBT facilitators were doctoral students majoring in clinical counseling. Both had received systematic coursework and supervised counseling practice. The MT facilitator had received formal training in mindfulness-based approaches and had three years of experience in mindfulness-related practice, whereas the CBT facilitator had received formal training in cognitive-behavioral approaches and had three years of experience in CBT-related practice.

### 2.4. Data Analysis

Data were analyzed using IBM SPSS 24 for descriptive statistics, *t*-tests and ANOVA analysis. Subsequently, we performed cross-lagged analysis using Mplus 8.3.

## 3. Results

A one-way ANOVA was first conducted to examine baseline differences among the three groups. The results showed no significant group differences at pretest for ADHD symptoms, F(2, 60) = 0.07, *p* = 0.932, $\eta_{p}^{2}$ < 0.01; test anxiety, F(2, 60) = 0.98, *p* = 0.38, $\eta_{p}^{2}$ = 0.03; or self-concept, F(2, 60) = 0.38, *p* = 0.689, $\eta_{p}^{2}$ = 0.01. These results are shown in Table 1.

### 3.1. Comparison of Intervention Effects

The pretest and posttest results for each variable across the three groups are presented in Table 2. To further evaluate the intervention effects, 3 (group: MT, CBT, Control) × 2 (Time: pretest, posttest) repeated-measures ANOVAs were conducted. For ADHD symptoms, the main effect of time was significant, F(1, 60) = 84.46, *p* < 0.001, $\eta_{p}^{2}$ = 0.59, indicating that posttest scores were significantly lower than pretest scores. The Group × Time interaction was also significant, F(2, 60) = 29.74, *p* < 0.001, $\eta_{p}^{2}$ = 0.5. Simple effects analyses showed that ADHD scores decreased significantly from pretest to posttest in the MT group, t = −8.26, *p* < 0.001, and in the CBT group, t = −9.27, *p* < 0.001. No significant pretest-to-posttest change was observed in the control group, t = 1.07, *p* = 0.866.

For test anxiety, the main effect of time was significant, F(1, 60) = 10.36, *p* = 0.002, $\eta_{p}^{2}$ = 0.15, with posttest scores significantly lower than pretest scores. The Group × Time interaction was also significant, F(2, 60) = 7.77, *p* = 0.001, $\eta_{p}^{2}$ = 0.21. Simple effects analyses indicated that test anxiety scores decreased significantly from pretest to posttest in the MT group, t = −3.1, *p* = 0.009, and in the CBT group, t = −4.1, *p* < 0.001. In contrast, the control group showed no significant change over time, t = 1.33, *p* = 0.568.

For self-concept, the main effect of time was significant, F(1, 60) = 59.67, *p* < 0.001, $\eta_{p}^{2}$ = 0.50, indicating that posttest scores were significantly higher than pretest scores. The Group × Time interaction was significant, F(2, 60) = 18.9, *p* < 0.001, $\eta_{p}^{2}$ = 0.39. Simple effects analyses showed that self-concept scores increased significantly from pretest to posttest in the MT group, t = 8.02, *p* < 0.001, and in the CBT group, t = 6.06, *p* < 0.001. No significant change was found in the control group, t = −0.47, *p* = 1.

The posttest scores for ADHD symptoms, test anxiety, and self-concept across the three groups of participants are presented in Table 3.

**Figure 1:**
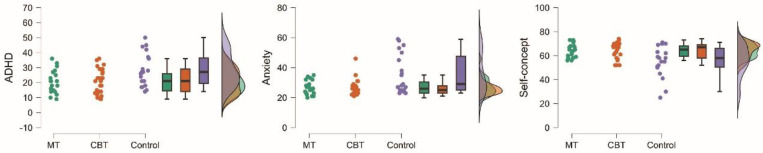
The differences in post-intervention scores on ADHD symptoms, test anxiety, and self-concept for the three groups.

To assess potential differences in intervention effects between the two methods, we performed one-way ANOVAs on posttest scores of ADHD symptoms, test anxiety, and self-concept for each group of participants. The results revealed a significant difference among the three groups in ADHD symptoms (F(2, 60) = 4.38, *p* = 0.02, *η*^2^ = 0.13). Post hoc comparisons indicated that the MT group’s scores were significantly lower than those of the control group (*p* < 0.05), and the CBT group’s scores were significantly lower than those of the control group (*p* < 0.05). However, there was no significant difference between the MT group and the CBT group. In terms of test anxiety, there was a significant difference among the three groups of participants (F(2, 60) = 8.54, *p* < 0.001, *η*^2^ = 0.22). Post hoc comparisons revealed that the scores of the MT group were significantly lower than those of the control group (*p* < 0.01), and the scores of the CBT group were significantly lower than those of the control group (*p* < 0.01). There was no significant difference between the MT group and the CBT group. Regarding self-concept, there was a significant difference among the three groups of participants (F(2, 60) = 6.27, *p* = 0.003, *η*^2^ = 0.17). Post hoc comparisons revealed that the scores of the MT group were significantly higher than those of the control group (*p* < 0.01), and the scores of the CBT group were significantly higher than those of the control group (*p* < 0.01). There was no significant difference between the MT group and the CBT group, as depicted in Figure 1.

### 3.2. Exploratory Cross-Lagged Associations

**Figure 2:**
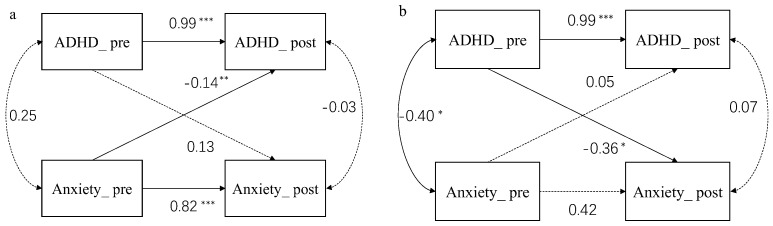
Cross-lagged analysis of ADHD symptoms and test anxiety between the MT group and CBT group. Note: (**a**) Mindfulness Training (MT) group. (**b**) Cognitive-Behavioral Training (CBT) group. Coefficients in the figure are standardized path coefficients. Solid lines represent significant path coefficients, and dashed lines represent non-significant path coefficients, * *p* < 0.05, ** *p* < 0.01, *** *p* < 0.001.

To explore potential patterns of association between ADHD symptoms and test anxiety in the two intervention groups, we conducted exploratory cross-lagged analyses using Mplus. The model estimation results for the relationship between ADHD symptoms and test anxiety are shown in Figure 2. The model was saturated. For the MT group, pretest test anxiety significantly negatively predicted posttest ADHD symptoms (β = −0.14, *p* < 0.01), while pretest ADHD symptoms did not significantly predict posttest test anxiety (*p* > 0.05). In contrast, for the CBT group, pretest ADHD symptoms significantly and negatively predicted posttest test anxiety (β = −0.36, *p* < 0.05), while pretest test anxiety did not significantly predict posttest ADHD symptoms (*p* > 0.05). For both the MT and CBT groups, the predictive effects between ADHD symptoms and self-concept were not significant in either the pretest or posttest (*p*s > 0.05).

## 4. Discussion

This study examined the effects of MT and CBT on ADHD symptoms, test anxiety, and self-concept in children with ADHD. The results showed that both MT and CBT were associated with significant reductions in ADHD symptoms and test anxiety and significant improvements in self-concept, whereas the control group showed no significant pretest-to-posttest changes. No significant posttest differences were found between the MT and CBT groups, suggesting that the two interventions produced broadly comparable improvements in the outcomes assessed in this study. Exploratory cross-lagged analyses showed different patterns of association between ADHD symptoms and test anxiety in the two intervention groups. However, these findings should be interpreted cautiously because the two-wave design cannot establish causal mechanisms.

Previous research has shown that MT may benefit individuals with ADHD, particularly in relation to attention, emotional regulation, and behavioral functioning (Cairncross & Miller, 2020; Canu et al., 2017; Lindstrom et al., 2020; Miller & Brooker, 2017). Although much of this evidence has come from adult samples, recent studies have also examined the use of MT in children with ADHD. For example, Siebelink et al. (2022) reported that MT was associated with improved attention in children with ADHD, which may have positive implications for academic functioning. In the present study, the 3 × 2 repeated-measures ANOVA showed that MT, like CBT, was associated with significant reductions in ADHD symptoms and test anxiety and improvements in self-concept. This finding is consistent with previous studies suggesting that non-pharmacological interventions may improve behavioral and emotional outcomes in children with ADHD (Hyseni Duraku et al., 2023; John Lothes et al., 2021; Lambez et al., 2020; Riise et al., 2021). MT emphasizes present-moment awareness and non-judgmental acceptance, which may help children respond to negative emotions in a more adaptive way (Fallah et al., 2023; Gan et al., 2023; Lindsay & Creswell, 2017).

The present study did not find significant posttest differences between the MT and CBT groups, suggesting that the two interventions were associated with broadly comparable improvements in the measured outcomes. Exploratory cross-lagged analyses were then conducted to examine preliminary patterns of association between ADHD symptoms and test anxiety in the two intervention groups. In the MT group, pretest test anxiety was associated with posttest ADHD symptoms, whereas in the CBT group, pretest ADHD symptoms were associated with posttest test anxiety. These findings may indicate different patterns of association between ADHD symptoms and test anxiety across the two interventions. However, because the cross-lagged analyses were based on only two time points, these results should be interpreted cautiously and should not be taken as evidence of causal mechanisms.

The Monitor and Acceptance Theory posits that MT comprises two essential components: attention monitoring and acceptance. Attention monitoring enhances cognitive abilities and emotional experiences, while acceptance reduces negative emotional experiences. The combination of these components effectively improves individual cognitive and emotional regulation capabilities (Gavrilova & Zawadzki, 2023; Lindsay & Creswell, 2017, 2019). The exploratory pattern observed in the MT group is broadly consistent with aspects of this theory. A recent meta-analysis showed that mindfulness interventions were associated with improvements in emotional outcomes such as anxiety and depression after controlling for publication bias and study quality (Johnson et al., 2023). The present findings are broadly consistent with this evidence.

In addition, our results revealed that CBT may primarily alleviate test anxiety by further ameliorating ADHD symptoms. Cognitive behavior theory indicates that cognitive adjustment and behavioral modification may improve the psychological well-being of individuals (Biesecker et al., 2017). A recent meta-analysis found that CBT yields the most substantial improvements in inhibitory control and externalizing issues (Lambez et al., 2020; Riise et al., 2021). The present findings are consistent with this theoretical perspective and were previously found.

This study developed an MT program tailored to the developmental characteristics of children with ADHD and found that it was associated with improvements in ADHD symptoms, test anxiety, and self-concept. The exploratory cross-lagged analyses showed different preliminary patterns of association between ADHD symptoms and test anxiety in the MT and CBT groups. In the MT group, pretest test anxiety was associated with posttest ADHD symptoms, whereas in the CBT group, pretest ADHD symptoms were associated with posttest test anxiety. These findings may provide preliminary evidence that the two interventions are linked to different patterns of change. However, they should not be interpreted as evidence of distinct causal mechanisms because the analyses were based on only two time points and relatively small intervention-group samples. Therefore, the present findings cannot determine which subtypes of children with ADHD may benefit more from MT or CBT. Future studies should examine whether children with different baseline symptom profiles respond differently to MT and CBT before drawing conclusions about personalized intervention matching. Our findings may also provide guidelines for classroom settings. This intervention can be operationalized in two ways. One is centralized intervention, where children with ADHD are gathered together for a unified intervention. The second is to integrate it into an actual classroom setting. In real classrooms, teachers can also draw on some of the operations in this study, such as integrating mindfulness breathing and body scanning into the teaching process to gradually improve students’ behavioral performance.

### Limitations and Future Research

The present study has several limitations. First, although we used a two-stage screening and eligibility confirmation procedure, participants were recruited from schools rather than clinical services. Future studies should replicate these findings in larger and more representative clinical samples. Second, the attrition rate during the eight-week intervention was relatively high, which may have introduced selection bias. Future research should record dropout reasons in greater detail and use appropriate missing-data methods. Third, full blinding was not feasible because of the behavioral nature of the intervention, and the control group continued regular classroom activities rather than receiving an active control intervention. Future studies should include blinded outcome assessment and active control conditions. Fourth, the intervention was delivered only to children with ADHD and did not include parent training, although previous studies suggest that combined child–parent interventions may produce stronger effects (Siebelink et al., 2022; Valero et al., 2022).

Several additional limitations should also be noted. First, the study assessed only immediate post-intervention outcomes and did not include follow-up assessments. Second, no a priori sample size calculation was conducted. Third, the cross-lagged analyses were based on only two time points and should therefore be interpreted as exploratory rather than as evidence of causal mechanisms. Future studies should include follow-up assessments, conduct a priori power analyses, and use more measurement points to verify and extend the present findings.

## Figures and Tables

**Table 1:** The demographic information of participants.

	MT (n = 19)	CBT (n = 25)	Control (n = 19)
Age	8.53 ± 0.61	8.40 ± 0.58	8.26 ± 0.56
Sex (% male)	58%	44%	58%
ADHD symptoms	26.32 ± 10.03	26.68 ± 11.49	27.58 ± 10.26
Test Anxiety	31.21 ± 6.83	31.85 ± 5.53	34.12 ± 8.10
Self-concept	55.32 ± 8.38	58.08 ± 12.43	56.00 ± 11.67

Note: MT = Mindfulness Training Group, CBT = Cognitive-Behavioral Training Group, Control = Control Group.

**Table 2:** ADHD symptoms, test anxiety, and self-concept scores for the three groups (M ± SD).

Variables	Pre			Post		
	MT	CBT	Control	MT	CBT	Control
ADHD Symptoms	26.32 ± 10.03	26.68 ± 11.49	27.58 ± 10.26	20.63 ± 7.8	21.12 ± 8.45	28.32 ± 11.05
Test Anxiety	31.21 ± 6.83	31.85 ± 5.53	34.12 ± 8.1	26.68 ± 4.7	26.64 ± 5.21	36.05 ± 13.05
Self-concept	55.32 ± 8.38	58.08 ± 12.43	56 ± 11.67	64.37 ± 5.67	64.04 ± 6.77	55.47 ± 13.21

**Table 3:** Post-intervention scores of the three groups on ADHD symptoms, test anxiety, and self-concept.

	MT (n = 19)	CBT (n = 25)	Control (n = 19)	F	*p*	*η* ^2^
ADHD Symptoms	20.63 ± 7.80	21.12 ± 8.45	28.32 ± 11.05	4.38	0.02	0.13
Test Anxiety	26.68 ± 4.70	26.64 ± 5.21	36.05 ± 13.10	8.54	<0.001	0.22
Self-concept	64.37 ± 5.67	64.04 ± 6.77	55.47 ± 13.21	6.27	0.003	0.17

## Data Availability

Data are available upon request.

## Supplementary Materials

The following supporting information can be downloaded at: https://www.mdpi.com/article/10.3390/bs16071128/s1, Table S1. The Contents of Mindfulness Training (MT); Table S2. The Contents of Cognitive Behavioral Training (CBT).
