# Supplementary material for: Eight-Week Mindfulness Training Effectively Improves Performance in Children with ADHD: A Comparison with Cognitive-Behavioral Training
Source: Behav Sci (Basel). 2026 Jul 6;16(7):1128. doi: 10.3390/bs16071128 (PMC13406042; doi:10.3390/bs16071128)
Supplement: Supplementary file 1 [file behavsci-16-01128-s001.zip › behavsci-4343034-supplementary.pdf]

Table S1. The Contents of Mindfulness Training (MT)

Week	Theme	Title	Objectives	Training Content
1	Understanding Mindfulness	Hello, mindfulness.	Understand what mindfulness is.	Introduce the basic content of mindfulness training.
2	Mindfulness Observation	I'm from Mars	Help children with ADHD to learn mindfulness observation.	Guide children with ADHD to imagine themselves as Martians and practice focusing their attention on raisins.
3	Mindfulness Breathing	Frog Sitting	Help Children with ADHD to learn mindfulness breathing.	Guide Children with ADHD to imagine themselves as frogs and practice sitting quietly, breathing like a frog.
4	Body Scan	Spaghetti Experiment	Help Children with ADHD to consciously relax their bodies.	Guide Children with ADHD to imagine their bodies as spaghetti and experience the transition from stiffness to relaxation.
5	Coping with Anxiety Emotion	Unpleasant Emotion First Aid	Help Children with ADHD to identify anxious emotion.	Teach Children with ADHD how to identify anxious emotion.
6	Coping with Anxiety Emotion	Anxiety Conveyor Belt	Help Children with ADHD to learn cope with anxious emotion.	Teach Children with ADHD to practice shifting attention away from anxious emotion.
7	Self-Acceptance	Unique Me	Help Children with ADHD to learn nonjudgmental self-acceptance.	Guide Children with ADHD to practice accepting themselves without judgement.
8	Self-Acceptance	My Future, My Choice	Review training outcomes, boost confidence, and enhance self-concept.	Allow Children with ADHD to share changes and growth after training, encouraging them to continue maintaining progress.

Table S2. The Contents of Cognitive Behavioral Training (CBT)

Week	Theme	Title	Objectives	Training Content
1	Understanding CBT	Hello, CBT.	Understand what CBT is.	Introduce the basic content of CBT.
2	Adjusting Cognition	Adjusting Cognition	Guide Children with ADHD to understand the importance of cognition.	Introduce the relationship between cognition and behavior.
3	Behavioral Training	My body, My Choice	Help Children with ADHD to understand hyperactivity symptoms.	Explaining the Symptoms of ADHD Hyperactivity and Using Situational Simulations to Help Children Feel the Impact of Hyperactivity on Their Attention.
4	Behavioral Training	My body, My Choice	Help Children with ADHD to learn control hyperactive and impulsive behavior.	Teach Children with ADHD behavioral training techniques, including breathing relaxation and not speaking exercises.
5	Coping with Anxiety Emotion	Recognizing Anxiety Emotion	Guide Children with ADHD to recognize anxiety emotion.	Introduce the relationship between emotion and behavior, allowing Children with ADHD to experience emotional responses under different behaviors.
6	Coping with Anxiety Emotion	Adapting to Anxiety Emotion	Help Children with ADHD to learn emotional coping strategies.	Teach emotional coping strategies, including emotional ABC training.
7	Self-Acceptance	Unique Me	Guide Children with ADHD to learn accept themselves.	Guide Children with ADHD to recognize their strengths and cultivate positive self-concept.
8	Self-Acceptance	My Future, My Choice	Review training outcomes, boost confidence, and enhance self-concept.	Allow Children with ADHD to share changes and growth after training, encouraging them to continue maintaining progress.
